# Should they stay or should they go? Negative interest rate policies under review

**DOI:** 10.1007/s10368-022-00547-4

**Published:** 2022-09-01

**Authors:** Joscha Beckmann, Klaus-Jürgen Gern, Nils Jannsen

**Affiliations:** 1 FernUniversität Hagen und Kiel Institute for the World Economy, Gebäude 3 (TGZ) Universitätsstraße 11, E-Trakt, 1. OG, 58097 Hagen, Germany; 2 Kiel Institute for the World Economy, Business Cycles and Growth, Kiellinie 66, 24105 Kiel, Germany

**Keywords:** Negative interest rates, Monetary policy, G12

## Abstract

Negative interest rate policies (NIRP) have become an established monetary policy instrument in the toolkit of the ECB. We discuss NIRP in the euro area based on theoretical considerations and available empirical evidence. We find that NIRP had some positive impact on loan growth and investment in the euro area, but that the room to further loosen monetary policy via NIRP may be small. NIRP is discussed also in the context of the general monetary policy environment.

## Introduction

In June 2014, the ECB was among the first major central banks to lower its policy rate into negative territory (-0.1% on the deposit facility rate). The deposit facility rate was subsequently reduced further four times – most recently in September 2019 to -0.5% – and the negative interest rate policy (NIRP) has become one among several unconventional tools in the ECB’s monetary policy toolkit. Unconventional measures, such as forward guidance and quantitative easing, were introduced because conventional interest policies were constrained by the zero lower bound. All of these measures can transmit via a set of various channels to financial markets, output, and inflation. Compared to other unconventional measures NIRP has a somewhat more direct link to banks and credit supply.

From a theoretical perspective, NIRP works similar to conventional interest rate cuts in positive territory. In principle, the transmission channels of interest rate cuts, for example via deposit and lending rate, to output and inflation do not change in negative territory. One key difference is, however, that holding cash, which offers a zero nominal return, becomes more attractive when interest rates are negative. While cash hording in theory lowers the impact of NIRP, there is no evidence that increases in cash holdings have significantly impeded the transmission mechanism of negative rates anywhere until now.

In practice, the difficulty for banks to charge negative rates on deposits of firms or private households weighs on bank lending and constrains the application of NIRP. If the pass-through to interest rates on deposits is weaker than to bank lending rates or to other assets of banks, NIRP compresses margins and weighs on bank profitability. Moreover, profitability of banks might be also negatively affected by payments they have to make to the central bank on excess reserves. The resulting reduction in banks’ net worth tends to reduce credit supply and, at some point, further interest rate cuts could become contractionary with respect to its effect on bank lending. This point is sometimes called the reversal interest rate, which represents the effective lower bound for NIRP. The ECB implemented measures to alleviate the negative impact of NIRP on bank profitability.

Entering negative territory with interest rates the first time can have a disproportional effect on expectations and risk-taking. If NIRP is not expected to be used by a central bank as an instrument, it will have more than proportional effects on expectations because it will shift the perceived lower bound for policy rates and provide a signal about the future stance of monetary policy. However, this is only a one-time effect that takes place when NIRP is implemented the first time. NIRP can also have a strong impact on risk-taking if economic agents switch to riskier assets just to avoid negative interest rates on their deposits due to psychological reasons. Higher risk-taking can amplify the impact of NIRP but is at the same time also a concern as excessive risk-taking can undermine financial stability.

In this paper, we discuss NIRP in the euro area based on theoretical considerations and the empirical evidence. In Section 2, we give a brief overview on the implementation of NIRP in the euro area in the context of other monetary policy measures. In Section 3, we briefly summarise monetary transmission channels from a theoretical perspective with a special focus on NIRP. In Section 4, we discuss the empirical evidence on the impact of NIRP on other interest rates and bank lending, bank profitability, risk-taking, and output and inflation. In Section 5, we conclude with a discussion of NIRP in the context of general findings on the impact of monetary policy on output and inflation, the natural interest rate, and potential negative side-effects.

## Extent, characteristics and duration of NIRP

**Fig. 1:**
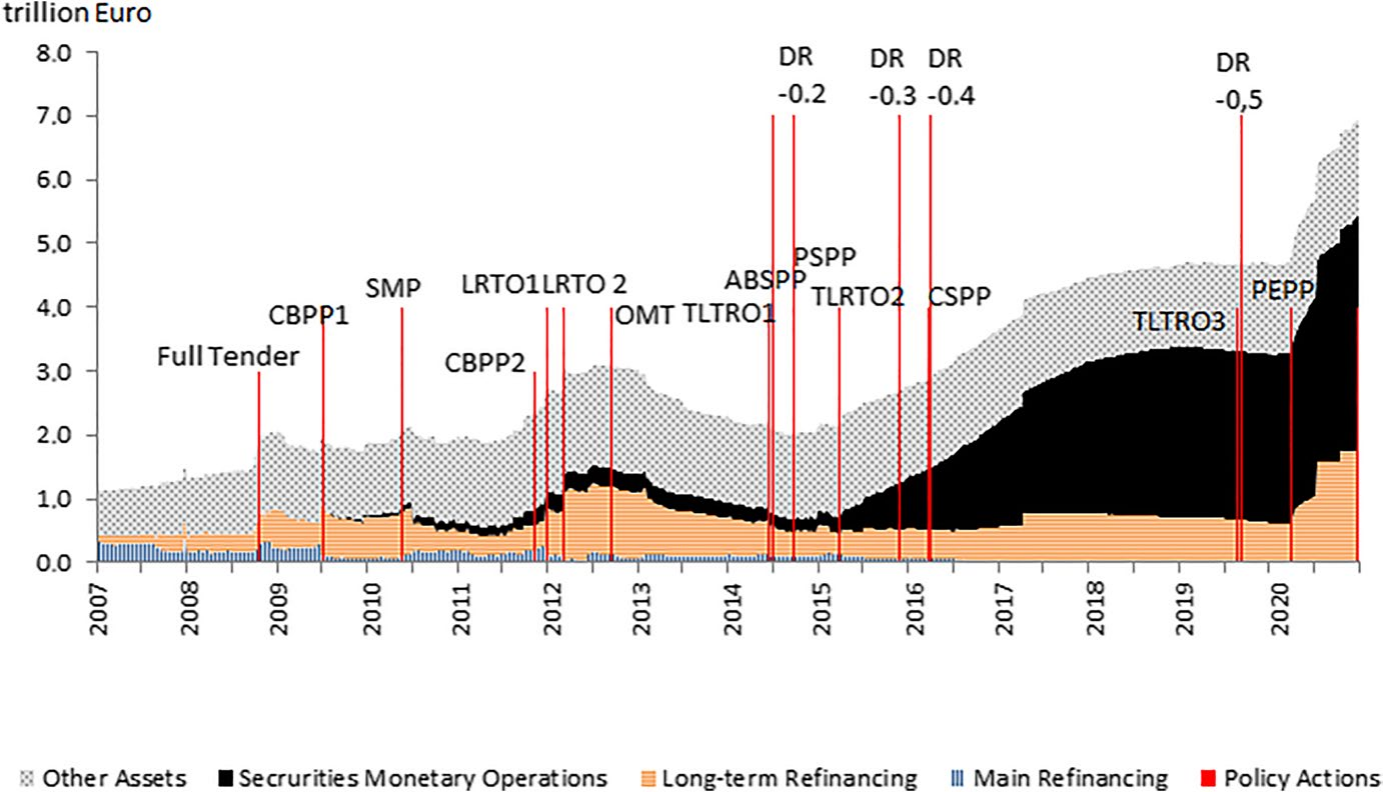
ECB balance sheet and monetary policy actions. Notes: “Securities Monetary Operations” includes securities purchase programmes; “Long-term Refinancing” includes ordinary 3-month operations and exceptional long-term refinancing operations and targeted longer-term refinancing operations (TLTROs); “Main Refinancing Operations” are ordinary operations that may be modified (e.g. fixed rate tenders with full allotment, reduced collateral requirements). “Policy Actions” depict the date of announcement of an action. “DR” denotes deposit rate; “SMP” denotes Securities Markets Programme; “CBP” denotes Covered bond purchase programme; “CSPP” denotes Corporate Sector Purchase Programme; “OMT” denotes Outright Monetary Transactions; ”PSPP” denotes Public Sector Purchase Programme; “PEPP” denotes Pandemic Emergency Purchase Programme. Sources: ECB, Eurosystem balance sheet, own calculations

Negative interest rates are part of the new monetary toolkit and have been introduced at a critical point for monetary policy in the euro area. Figure 1 puts negative interest rates in a broader context with regard to other measures based on an assessment of the ECB balance sheet and specific monetary policy actions, including shifts in the deposit rate, which are illustrated as red bars. The ECB shifted its lending policy strongly towards long-term refinancing operations from 2009 onwards, enabling banks to receive liquidity for a period of up to 3 years, whereas main refinancing operations only provide liquidity for one week. Around the same time, other assets and securities from monetary operations started to increase due to asset purchases as a result of the quantitative easing policy. These policies resulted in a significant amount of excess reserves in the banking sector. Negative interest rates were introduced to complement these measures with the aim of boosting credit and increasing inflation expectations. As discussed in Section 4, it is, however, difficult to identify additional effects of negative interest rates with regard to macroeconomic outcomes.

The ECB policy has linked the exposure to negative interest rates to the business models of banks. On 30 October 2019, the ECB introduced a new remuneration structure with two distinct rates applicable to different parts of the excess reserve holdings of credit institutions. The two-tier system exempts credit institutions from remunerating, at the negative rate currently applicable on the deposit facility, a part of their excess reserve holdings depending on minimum reserve requirements, which are mainly based on bank customers’ deposits. The two-tier system therefore effectively reduces payments to the ECB on excess reserves for banks whose business models rely on deposit funding, which are typically the main lenders to the real economy in the euro area.

The actual exposure of banks to negative rates is also reduced by the design of targeted long-term refinancing operations (TLTROs). A first series of TLTROs was announced on 5 June 2014, a few days before the deposit facility rate was moved into negative territory on 11 June 2014. The introduction of TLRTO III closely coincided with the reduction of the deposit facility rate to -0.5%. The interest rate for TLTRO III borrowings is linked to the deposit facility rate and depends negatively on the amount of loans participating banks grant to non-financial corporations and households. Borrowing rates for TLTRO operations can be as much as 50 basis points below the average interest rate on the deposit facility over the period from 24 to 2020 to 23 June 2022, and as low as the average interest rate on the deposit facility during the rest of the life of the respective TLTRO III.

**Fig. 2:**
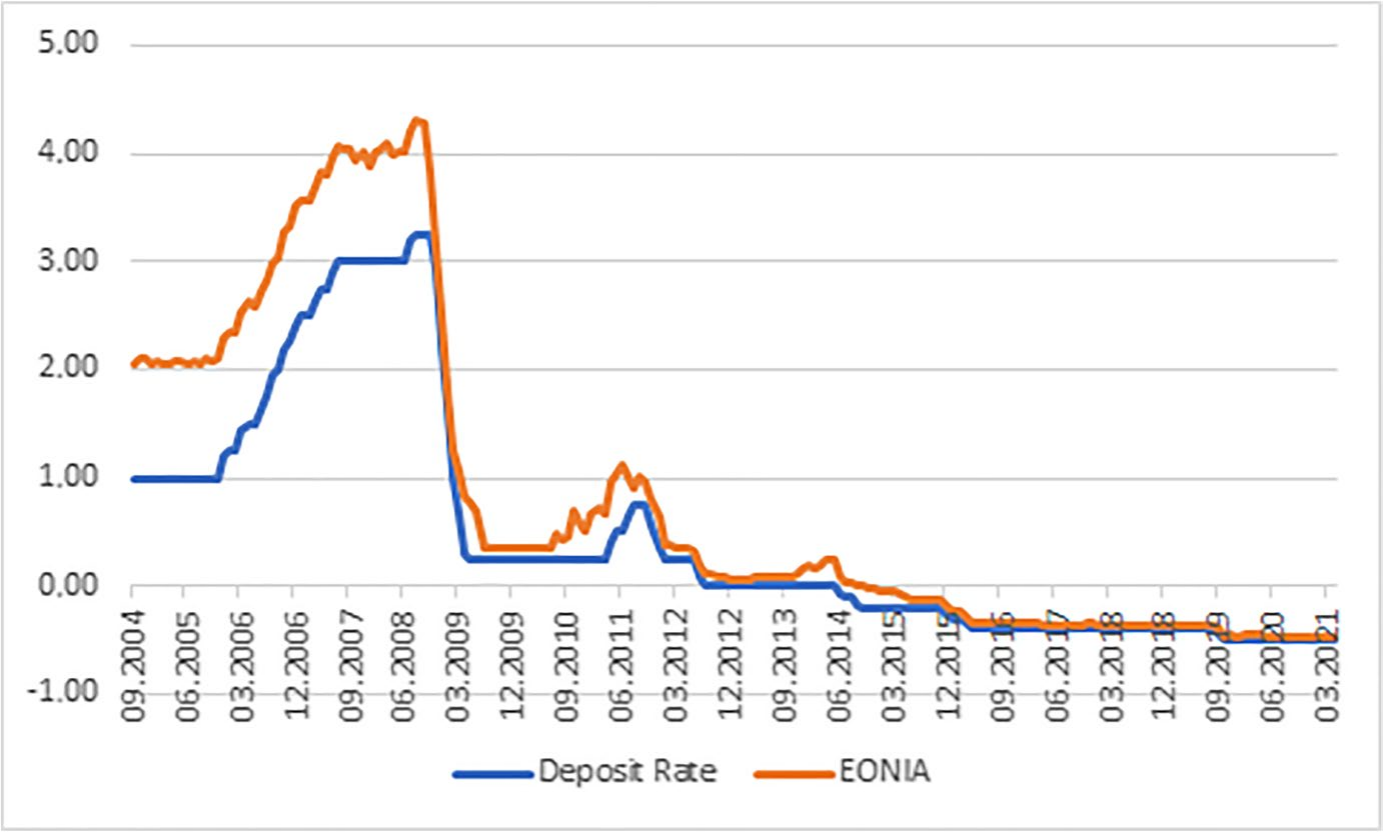
Deposit rate and EONIA. Source: Own illustration based on ECB data

The negative deposit facility rate has been transmitted to interbank loan rates. Figure 2 illustrates that the Euro Overnight Index Average (EONIA) followed the deposit rate into negative territory. The EONIA was closer linked to the main refinancing rate until 2014, suggesting the deposit rate acts as a close substitute since the main refinancing rate is stuck at the zero lower bound.

## Theoretical effects of NIRP

Negative interest rates can be transmitted via different channels to the economy related to both conventional and unconventional monetary policy measures. Compared to conventional interest rate changes, some of the transmission channels might be weaker under negative interest rates while others remain intact or might be even stronger from a theoretical perspective. In this section, we focus on the corresponding propagation mechanisms that are particularly relevant for NIRP. We discuss the interest rate channel, the bank lending channel, the balance sheet channel, the risk-taking channel, and the expectation channel. We also briefly summarise the exchange rate channel but do not discuss details in this paper. Given the focus on the empirical evidence on the effects of NIRP in this paper, in the rest of this section we discuss different aspects of bank lending and the reversal interest rate, risk-taking and expectations related to NIRP in greater detail.

The interest rate channel, which describes the pass-through of lower policy rates to consumers and firms, can be weaker under negative interest rates. The profitability of banks depends, among other factors, on the spread between interest rates to loans and deposits. If banks are reluctant to pass-through interest rates to deposit rates of their clients, in particular to households, they are also less likely to reduce interest rates to loans in order to protect their interest rate margin. This would lower the impact of NIRP because consumption or investment decisions by private households are less stimulated by lower interest rates compared to conventional interest rate cuts in positive territory.

The bank lending channel describes how monetary policy affects credit supply of banks and is particularly relevant for NIRP. Via the bank lending channel monetary policy can influence the credit supply of banks and thereby spending and investment decisions of firms. Expansionary monetary policy tends to increase the credit supply of banks. This effect can even be stronger under negative interest rates since banks have stronger incentives to reduce their excess reserves by increasing their credit supply. However, the concept of a reversal interest rates emphasises that at some point negative interest rates can dampen credit supply of banks if they hurt bank profitability. Bank profitability can be dampened by NIPR if—in addition to the payments banks have to make for their excess reserves—banks cannot pass through negative interest rates to the same extent to deposit rates as to lending rates lowering their interest rate margin. If these negative effects outweigh the positive effects on bank profitability, e.g. due to the increase in the value of long-term mark-to-market assets or higher credit volumes, further interest rate cuts can lower credit supply and thereby counteract the intended effect of NIRP. The response of banks to negative interest rates can depend on individual characteristics, such as the reliance on deposits or the volume of excess reserves. Understanding the response of banks to NIRP is therefore one key element for assessing the effects of NIRP.

The balance sheet channel describes that changes in the monetary policy stance affect also credit demand. The interplay of bank lending channel and the balance sheet channel constitutes the credit channel of monetary policy. Monetary policy can affect the financial position of borrowers and thereby credit demand. Expansionary monetary policy decreases the interest rate expenses on their short-term debt, which improves their financial positions. Lower interest rates can also lead to an increase in asset prices. This increases the net worth of firms and households and stimulates credit demand via (i) an improved access to bank loans, and (ii) an improved economic outlook and higher willingness to invest. The balance sheet channel does not necessarily work differently under negative and positive interest rates since asset prices are potentially affected in both scenarios by interest rate cuts.

The risk-taking channel, which describes the effect of monetary policy via influencing risk-taking by banks, firms, or private households could be stronger under negative interest rates. If “banks’ excess reserves are remunerated at negative rates, there is a strong incentive to reduce them by shifting into riskier assets. This strengthens the portfolio rebalancing channel of asset purchases” (Schnabel 2020). Firms or private households might also shift to riskier assets to avoid negative interest rates on deposits if banks pass-through negative rates.

Negative interest rates are a commitment to low interest rates for the foreseeable future and can also have an impact on expected inflation. Negative interest rates can complement unconventional monetary policy measures such as signalling and forward guidance by affecting expectations regarding the future stance of monetary policy. The impact on expectations could be particularly strong when a central bank implements negative interest rates for the first time if the implementation affects expectations on the non-negativity restriction on current and future expected short-term rates and, therefore, monetary accommodation can propagate throughout the yield curve (Boucinha and Burlon 2020). However, maintaining negative interest rates can also send a signal about the future stance of monetary policy and thereby affect expectations.

The exchange rate channel of monetary policy is more relevant for more open economies, such as Sweden, Denmark, Norway and Switzerland compared to the euro area or the US. Lower domestic interest rates make the currency less attractive to foreign investors and reduce capital inflows. The subsequent depreciation (or prevention of further appreciation) makes domestic goods cheaper internationally and thus increases sales potential, which supports economic growth, and increases prices for imported goods. However, this line of reasoning is based on the assumption that monetary policy in other regions does not react to exchange rate movements. Overall, exchange rates are affected by a rich set of determinants and difficult to predict.

### Bank lending and reversal interest rate

Negative interest rates have made central bank deposits less attractive and increased the incentives for banks to raise credit supply. Quantitative easing and long-term refinancing operations have led to a strong increase of excess reserves of banks. In particular under negative deposit rates, banks have incentives to pass on central bank deposits (“hot potato effect”) that are not necessary for minimum reserve requirements (Ryan and Whelan 2021). One way to reduce excess reserves is to increase lending to firms or private households. However, banks can also reduce excess liquidity by purchasing debt securities of other banks or by paying down funding sources.

The “reversal interest rate” defines the level of interest rates at which monetary policy reverses its intended effect and becomes contractionary for lending due to negative effects on bank profitability. It occurs when the positive effects of lower interest rates on banks’ profitability and balance sheets, e.g. due to asset revaluation, are more than offset by negative effects, e.g., due to decreases in interest rate margins. Conceptually, the reversal rate reflects an effective lower bound for monetary policy, at which further interest rate cuts become ineffective or even counterproductive.

Brunnermeier and Koby (2018) identify four determinants of the level of the reversal interest rate: (1) banks’ fixed-income holdings, (2) the strictness of capital constraints, (3) the initial capitalisation of banks, and (4) the degree of pass-through to deposit rates. A large proportion of banks’ fixed-income holdings provide stable earnings even if central bank interest rates fall. Therefore, the larger the share of fixed income holdings the lower the reversal rate. Low initial capitalisation or tight capital requirements, e.g. from banking regulation, reduce the flexibility of banks to adjust to smaller interest rate margins due to a lack of free capital. This increases the reversal rate. The reversal rate also increases if the interest rate elasticity of customer deposits is very high, so that interest rate cuts by the central bank are more difficult to be passed on to customers.

The reversal rate increases over time (“creeping up effect”) implying that prolonged negative interest rates increase the probability of adverse effects on lending. Interest rate cuts lead to increases in the price of assets, thus strengthening balance sheets of banks and increasing their credit supply (bank capital channel). However, the longer interest rates remain low, the lower interest income on new assets becomes so that the positive effect becomes smaller over time and could eventually reverse even if interest rates are not lowered further. Quantitative easing tends to increase the reversal interest rate over time since long-term income holdings of banks are reduced (Brunnermeier and Koby 2018). However, Repullo (2020) argues that lower negative rates will not necessarily at some point become contractionary for lending. He argues that profitability constraints of banks usually do not lead to a reversal rate since lower policy rates always increase the banks’ profits from lending given that they reduce the weighted average cost of deposits and capital. For the same reason, with a downward sloping demand for loans, they increase bank lending.^1^

### Risk-taking

Academic research has emphasised the importance of a risk-taking channel of monetary policy in the context of credit demand and supply. Uncertainty in the banking sector restricted credit supply after the 2008/2009 crisis since banks were reluctant to take on risks (Paligorova and Jimenez 2012). Consequently, one aim of central bank policies has been to encourage risk-taking. However, excessive risk-taking undermines financial stability. Therefore, macroprudential regulation is important in the context of the risk-taking channel in order to reduce the risk of excessive risk-taking (Borio and Zhu 2008).

The link between monetary policy and the perception and pricing of risk by economic agents is also important in order to understand the specific effects of negative interest rates. Two channels are particularly relevant: (1) Lower interest margins by banks could lead banks to take higher risks in lending in order to obtain higher risk premiums, and (2) A “search for yield” could lead to an increasing demand for assets with higher expected returns. The first effect materialises through the lending structure of banks while the second mechanism affects asset prices.

Bank characteristics, such as the structure of balance sheets, bank profitability and the ability to pass-through, determine whether NIRP have large effects on risk-taking behaviour. Banks which are able to pass-through negative interest rates to deposits do not experience lower interest rate margins and have therefore less incentives to take higher risks, while banks which experience a reduction in margins are more likely to take on additional risks.

### Expectations

Along with other monetary policy measures, negative interest rates were also implemented to increase inflation expectations and reduce expected real interest rates. By lowering the perceived lower bound for interest rates, central banks can send a signal that short-term interest rates will stay low (or negative) for the foreseeable future. The possibility for the market to anticipate further policy cuts is a fundamental element of monetary policy, and negative interest rates have also been a communication tool which can result in substantial revisions in macroeconomic expectations of households and firms. These revisions in expectations can influence future macroeconomic outcomes.

When NIRP is implemented for the first time, the zero lower bound is no longer constraining market expectations. Therefore, investors’ demand for longer-dated assets is increasing more than when rates are cut in positive territory (Tenreyro 2021). The question is whether such effects on expectations are a one-off effect once the negative territory is entered.

The signaling channel lowers expectations of future short-term interest rates and these expectations are directly linked to long-term interest rates and the yield curve (Groot and Haas 2019). Consequently, monetary policy effects on output and prices through expectations are frequently assessed via yield-curve dynamics. Theoretical and economic evidence suggests that the slope of the yield curve is positively related to economic activity and bank profitability. For the transmission of QE to yield curve dynamics, the importance of the portfolio rebalancing channel and the signaling channel has been particularly emphasised in the literature (Altavilla et al. 2015). The portfolio rebalancing channel affects the risk premium and works if short-term and long-term bonds are imperfect substitutes, so that the purchase of long-term government bonds changes the relative supply of short and long bonds so that the risk premium is reduced (Gagnon et al. 2010).

## Empirical evidence on effects of NIRP in the euro area

### Pass-through of negative interest rates and bank lending

The empirical evidence shows that the pass-through of negative interest rates to loan rates is incomplete. Loan rates to corporates are partly reduced, while mortgage rates are often not affected or even increased. Table 1 summarises the empirical evidence on pass-through to lending rates and also mentions how the exposure of banks to negative interest rates is identified based on micro level bank data. This is usually done according to the amount of deposits subject to negative interest.

**Table 1:** Pass-through evidence

	Author	Data / Identification	Results for banks stronger exposed to negative interest rates
Euro area	Amzallag et al. 2019 (Italy)	Mortgage-backed securities and confidential data on mortgage loans / Overnight deposits of banks	Higher fixed mortgage rates (1/4 of total) but similar floating rates
	Bottero et al. 2019 (Italy)	Interbank loans & securities based on credit register data / Retail deposit of banks	Lower corporate loan rates
	Tan 2019	Individual balance sheet items (IBSI) and individual MFI interest rates (IMIR) / Deposits	Lower mortgage spreads (limited evidence)
Sweden	Eggertsson et al. 2019	High-frequent microdata on bank-level interest rates / Event study based on deposit rate	Less mortgage rate pass-through
Denmark	Adolfsen and Spange 2020	Deposits / Event study based on deposit rate	Similar pass-through to households & corporates (but slower than normal)
Switzerland	Basten and Mariathasan (2018)	Supervisory information / Fraction of central bank reserves exempt from negative rates	Higher mortgage rates
	Schelling and Towbin (2020)	Micro data set on individual Swiss corporate loans / Deposits and reserves	Lower corporate loan spreads, lower or similar mortgage spreads

Own illustration based on Tenreyro (2021)

Empirical studies have shown that (with few exceptions such as Switzerland) a zero lower bound applies to household deposits. This reduces the amount of pass-through for banks which rely on deposit from private customers.

Private households tend to perceive negative interest rates on deposits as unnatural and may withdraw their deposits, notwithstanding of the associated costs of holding cash and making payments. A survey of 13,000 bank customers in Europe, the US and Australia by ING in 2006 found that about 77% of respondents would liquidate their deposits if interest rates went into negative territory, although with wide variations between countries. The authors attribute this to the behavioral economics concept of loss aversion: subjects feel losses twice as intensely as gains of the same amount. Consequently, an interest rate cut from 0 to -0.5% hurts significantly more than an interest rate cut from + 1.0 to + 0.5%. In some countries, around 50% of respondents said they would rather hoard cash than pay negative interest rates. However, the negative interest rate policy, which has already been in place for five years, may lead to a habituation effect that gradually lowers customer resistance. For example, monthly fees were initially increased on checking accounts that were largely free of charge just ten years earlier, which ultimately also meant a kind of negative interest rate for customers. There is also anecdotal evidence that negative interest rates on household deposits are being phased in in more and more banks in countries like Denmark (Krogstrup et al. 2020) and Germany (Tagesschau 2020).

**Fig. 3:**
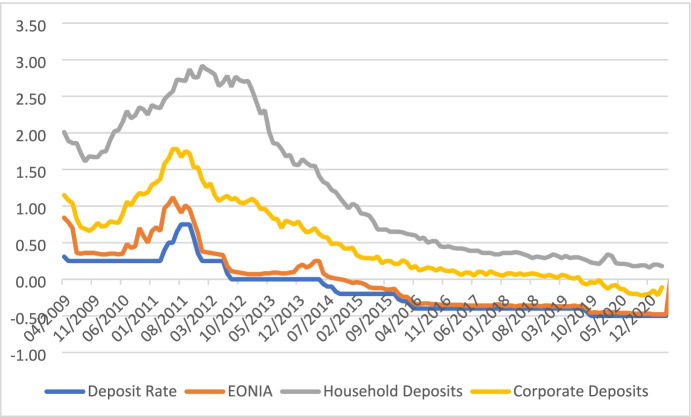
Household and corporate deposit rates, up to one year. Source: Own illustration based on ECB data

For deposits from business customers, on the other hand, negative interest rates on deposits seem easier enforceable. Figure 2 illustrates that rates on corporate deposits in the euro area have already entered negative territory, while household deposits seem restricted at close to zero. Owners of larger volumes of liquid assets, especially businesses, cannot convert to cash and will place their money with the most stable and trustworthy banks. Altavila et al. (2019) report that on average 20% of corporate deposits are subject to negative interest rates, but only 5% of total deposit volumes. Based on confidential data from 2017 to 2018, they find that sound banks pass on negative rates to their corporate depositors without experiencing a contraction in funding (Fig. 3).

The evidence supports the proposition that the ability to pass through negative interest rates and their effect on profitability depends on the deposit structure of banks. Banks with higher customer deposits are stronger exposed to negative interest rates compared to low-deposit banks, which reduces their income resulting from interest rate spreads (Heider et al. 2019). Evidence based on deposits, self-reported impact and reserves clearly suggests that banks tend to increase loans stronger if they are more exposed to negative interest rates. This holds for household and corporate lending while the evidence for syndicated lending is inconclusive. Table 2 summarises the empirical evidence with regard to data, identification and main results.

**Table 2:** Bank lending evidence

	Author	Data / Identification	Results for banks stronger exposed to negative interest rates
Euro area	Arce et al. (2018)	Survey data and credit register / Self-reported NII impact	Similar corporate lending but possible negative effect over the long-run
	Altavilla et al. (2018)	Proprietary and commercial data on individual euro area bank balances / Self-reported NII impact	More corporate lending
	Bittner et al. (2020) (Germany & Portugal)	Credit-registry data from Germany and Portugal / Deposits	Mixed results for corporate lending
	Bottero et al. (2019) (Italy)	Interbank loans & securities based on credit register data / retail deposit of banks	More corporate lending
	Bubeck et al. (2020)	Securities register for the 26 largest euro area banking groups / Deposits	Increase securities holdings; mixed evidence on syndicated loan volumes
	Demiralp et al. (2019)	Banks’ funding structures and excess liquidity holdings / Deposits and reserves	More household and corporate lending for high deposit banks; similar private securities holdings
	Grandi and Guille (2021) (France)	Bank firm level data / Deposits	More lending, especially to corporates, increase dept securities holdings
	Heider et al. (2019)	Syndicated loans and firm data / Deposit to asset ratios	Less syndicated lending
	Klein (2020)	Proprietary bank-level data / Impact of lower net interest rate margin	Usual hit to lending vanishes under NIRP
	Tan (2019)	Individual balance sheet items (IBSI) and individual MFI interest rates (IMIR) / Deposits	More lending (though effect dissipates), driven by mortgages, similar corporate lending
Sweden	Eggertsson et al. (2019)	High-frequent microdata on bank-level interest rates / Event study based on deposit rate	Less household lending
Denmark	Adolfsen and Spange (2020)	Deposits / Event study based on deposit rate	Stronger or similar lending to households & corporates (depending on specification)
Switzerland	Basten and Mariathasan (2018)	Supervisory information / Fraction of central bank reserves exempted from negative rates	Increase in share of loan assets (both uncollateralised loans and mortgages)
	Schelling and Towbin (2018)	Micro data set on individual Swiss corporate loans / Deposits and reserves	More corporate lending
Japan	Hong and Kandrac (2018)	Equity price response to NIRP announcement	More lending

Own illustration based on Tenreyro (2021)

Banks with a high proportion of private customer deposits also grant more credits under negative interest rates. The inability to pass through negative interest rate reduces margins and profits and brings up the need to reduce negative interest excess reserves. Banks with a high proportion of deposits increased their lending by 8.1% as a result of the NIRP, while banks with a medium proportion of customer deposits on their balance sheet increased their lending by only 2.8% (Demiralp et al. 2019). However, evidence also suggests less lending by euro area banks with a greater reliance on deposit funding with regard to syndicated loans (Heider et al. 2019). Evidence from proprietary bank-level data also shows that the link between the net interest margin and lending changes when interest rates become negative. When lending is less profitable, banks normally cut lending. However, at negative rates this is no more the case. This finding suggests that banks adjust their business practices when providing new loans, thereby contributing to higher new lending in the euro area since 2014 (Klein 2020). On the other hand, evidence for Denmark, Sweden and Switzerland suggests that negative interest rates did not have a significant effect on bank lending growth or inflation in any of these countries (Michail 2019).

It is not straightforward to derive lessons for the ECB from experience in other countries which implemented NIRP given differences in the banking structure and different monetary policy aims. Denmark and Switzerland frequently intervene in the foreign exchange market and adopted negative interest rates also to prevent capital inflows and appreciation pressure on the domestic currency. Negative interest rates are considered to have been effective in lifting inflation and inflation expectations in Sweden and in stabilising the exchange rate in Denmark, with banks’ profitability continuing to improve (Madaschi and Nuevo 2017). However, given the structural differences with regard to the importance of negative interest rates, these findings do not necessarily mitigate concerns for the euro area. Evidence for Switzerland points to negative effects on bank profitability, and Swiss banks have increased their pass-through over time. Overall, a common pattern is that all central banks have eased the burden of negative interest rates via the allowance of reserves free of charge.

### Bank profitability and the reversal interest rate

Preliminary international evidence suggests that negative interest rates have no major negative side effects on bank profits, payment systems, and market functioning (IMF, 2017). For the euro area, there is no conclusive evidence that negative rates have hurt overall bank profitability. Banks seem to have offset interest income losses under negative rates with lower deposit expenses and gains in non-interest income, including fees and capital gains. Small banks and low-deposit banks drive most results (Lopez et al. 2020). Altavilla et al. (2019) analyse data from 2000 to 2016 and show that monetary policy easing – a decrease in short-term interest rates and/or a flattening of the yield curve does not reduce bank profits once the endogeneity of policy measures is accounted for. While the existing literature for the euro area all in all does not point to negative effects on bank profitability, the studies generally cover only the early years of NIRP and are thus not necessarily informative with regard to negative effects of maintaining negative interest rates over an extended period of time. Table 3 summarises the existing literature and illustrates the lack of evidence for the most recent years. There is also the open question how the effects of negative interest rates and the effects from the pandemic combine.

**Table 3:** Bank profitability evidence

	Author	Data	Results
Euro area	Ampudia and Van den Heuvel (2018)	Intraday (tick-by-tick) data on swap rates, sovereign bond yields and individual bank stock from 1999–2017	Interest rate cuts in negative territory have a negative effect on banks’ equity values which is much more pronounced for banks with a high reliance on deposit funding
	Arce et al. (2019)	Individual balance sheets (IBSI) of a sample of 122 banks, for 13 euro area countries from 2014 Q2 to 2017 Q	Banks that report an adverse effect of negative interest rates on their net interest income have lower average capital ratios
	Altavilla et al. (2018)	Proprietary data on individual euro area bank balance-sheets and market prices from Q1 2007–Q4 2016	Monetary policy easing does not compress bank profits, but being exposed to a low interest rate environment for a protracted period might exert downward pressure on bank profitability
	Boucinha and Burlon (2020)	Net interest income of 194 euro area banks	Broadly neutral impact on bank profitability
	Carbó-Valverde et al. (2021)	Dataset on interest rates on bank margins of 3,155 banks from 36 European countries over the 2011–2018 period	Banks in negative interest rate environments experience a 17.4% decrease in their net interest margins compared to other banks. Negative effects more pronounced for banks with high customer deposits
	Heider et al. (2019)	Syndicated loans granted by euro area banks between 2011 and 2015	High-deposit banks have to deal with a reduction in their net worth under negative interest rates
	Stráský and Hwang (2019)	50 banking groups directly supervised by the Single Supervisory Mechanism from 2014–2018	Weak evidence of possible negative effects on bank profitability
International evidence	Beauregard and Spiegel (2020)	Annual data from 27 European countries and Japan from 2010 through 2018; accountability measure	Persisting negative rates have negative effects on bank profitability
	IMF (2017)	Case studies for euro area, Japan, Sweden, Denmark Switzerland	Limited negative side effects on bank profitability
	Lopez et al. (2020)	Cross-country panel of over 5,100 banks in 27 countries from 2010–2016; accountability measure	Heterogeneous effects of negative rates, low-deposit banks have enjoyed gains in non-interest income. Banks responded to negative rates by increasing lending activity, and raising the share of deposit funding. Overall benign implications of negative rates for commercial banks
	Molyneux et al. (2019)	7,359 banks from 33 OECD member countries over 2012–2016	Bank margins and profits fell in countries with negative interest rates relative to other countries- adverse. Effects depends on bank specific-characteristics such as size, funding structure and business models
Sweden and Denmark	Madaschi and Nuevo (2017)	Net interest income margin; realised and unrealised gains on securities	Profitability of banks in Sweden and Denmark has continued to improve und negative interest rates
Japan	Hong and Kandrac (2018)	Balance sheet and income statement from banks from 2015–2017	Sharp drop in equity prices of Japanese financial firms after announcement.of negative interest rates
Switzerland	Ziegler-Hasiba and Turnes (2018)	Literature survey of 97 articles with a focus on Switzerland	Lower net income of Swiss banks from the commission and service business

Own illustration

Effects of negative interest rates on bank profitability are measured via the reaction of banks’ stock prices to policy rate announcements, the effect on net interest income, or accounting-based measures. Interest rate reductions tend to have a positive effect on bank profitability in normal territory but the evidence suggests that this effect may be reversed under negative rates. Bank stock prices do for example respond positively to lower policy rates in positive territory but negatively in negative territory. Accounting-based measures tend to be more backward looking and are available only at lower frequency. International evidence based on such measures suggests that net interest margins and overall profitability in OECD countries is negatively affected by the introduction of negative rates (Molyneux et al. 2019). However, banks are frequently able to compensate these losses via increases in non-interest income.

Effects on bank profitability depend on various factors and some banks might come under substantial pressure, in particular if negative rates persist. Stock prices of banks with high dependence on deposit funding react particularly negatively to a negative policy rate (Heider et al. 2019; Ampudia and Van den Heuvel 2018) find that “an unexpected decrease of 25 basis points on the short-term policy rate increases banks’ stock prices by about 1% on average” while interest rate cuts in negative territory have a negative effect on banks’ equity values. “The change in sensitivity to interest rate surprises as rates drop to low and negative levels is much more pronounced for banks with a high reliance on deposit funding”,^2^ Similar results are obtained if accounting-based measures for profitability are adopted (Lopez et al. 2020).

Contractionary effects from negative interest rates on bank profitability have been partly compensated by recent policy measures. The adoption of the two-tier system through which reduces the amount of excess reserves subject to negative rates and TLTROs which link interest rates to the amount of credit is a sensible measure since it allows banks to adjust to the new environment. This is also in line with Darracq Pariès et al. (2020) who emphasise complementarities between monetary policy and macroprudential policy since the risk of hitting the reversal rate depends on the capitalisation of the banking sector. They find that macroprudential policy in the form of a countercyclical capital buffer can mitigate the probability of encountering the reversal rate which they locate in negative territory of around − 1% per annum.

### Risk-taking behaviour

The empirical evidence suggests that banks more exposed to negative interest rates are willing to take up higher risk. Table 3 summarises the corresponding evidence with regard to risk-taking of banks (Table 4).

**Table 4:** Risk-taking evidence

	Author	Data	Results
Euro area	Arce et al. (2018)	Survey data and credit register / Self-reported NII impact	Similar corporate loan standards, lower risk tolerance and risk weighted assets
	Bittner et al. 2020) (Germany & Portugal)	Credit-registry data from Germany and Portugal / deposits	Mixed results, but more credit to new risky firms in Germany
	Bottero et al. (2019) (Italy)	Interbank loans & securities	Bigger rise in lending for risky firms
	Bubeck et al. (2020)	Securities register for the 26 largest euro area banking groups / Deposits	Buy riskier securities, increase syndicated lending for riskier borrowers
	Heider et al. (2019)	Syndicated loans and firm data / deposit to asset ratios	Increase in average borrower risk
Switzerland	Basten and Mariathasan (2018)	Supervisory information / Fraction of central bank reserves exempted from negative rates	Higher risk-weighted asset share
	Schelling and Towbin (2018)	Micro data set on individual Swiss corporate loans / Deposits and reserves	More risk in corporate lending (across various dimensions)
Japan	Hong and Kandrac (2018)	Equity price response to NIRP announcement	Higher loan portfolio yield, interpreted as higher risk borrowers, and longer loan maturities

Own illustration based on Tenreyro (2021)

Overall, the effect of negative interest rates on risk-taking of banks is heterogeneous and depends on the funding structure. The overall effects of interest rate changes on risk-taking is ambiguous under positive interest rates, but there is strong evidence that negative interest rates tend to increase risk-taking.

“High-deposit banks tend to increase their holdings of high-yield securities in an environment of negative deposit rates, especially relative to low-deposit banks” (Bubeck et al. 2020). Risk-taking is also closely related to the ability to pass through negative interest rates to deposit rates. Heider et al. (2019) examine data at the individual loan level for syndicated loans. They find that banks with a high share of customer deposits (since the start of NIRP) reduced their lending volumes to safe borrowers and expanded the volumes extended to riskier borrowers.

Risk taking can amplify positive effects on economic activity. Borrowers of high-deposit banks in Germany are for example “riskier but they increase investment and employment more strongly after receiving credit, thereby supporting monetary transmission to the real economy” (Bittner et al. 2020).

Risk-taking can pose a risk to financial stability, if lending is done by under-capitalised banks. So far, there is little evidence for unintended side effects in the form of excessive growth of house prices, higher stock market volatility or excessive credit growth (Beck et al. 2019). However, it is difficult to distinguish between appropriate and excessive risks in real time, and policies cannot be adjusted overnight. Adequate macroprudential policies are therefore important to prevent negative effects stemming from risks, bearing in mind that too restrictive actions could also reduce potential positive effects from risk taking.

### Impact on expectations, output and inflation

Quantifying the impact of NIPR on output and inflation is very difficult due to the multiple transmission channels and the due to different unconventional monetary policy measures that were implemented in the same period. As a consequence, the empirical literature on the effects of unconventional monetary policy measures and in particular of NIRP on output and inflation is scarce. Some studies try to assess the impact of NIRP on output with firm-level data. However, it is difficult to draw conclusions from firm-level data to the overall macroeconomic impact. Other studies try to identify the impact of unconventional policy measures on financial market variables and then use counterfactual experiments to draw conclusions for different macroeconomic variables. However, the estimation uncertainty in such experiments is high.^3^ One reason is that the relationship between financial market variables and macroeconomic variables, such as output and inflation, can depend on the economic circumstances and can vary over time, which is difficult to account for.

Firm-level evidence suggests that NIRP can lead to an increase of investment and employment. Bittner et al. (2020) find that banks in Germany, which are particularly exposed to NIRP due to high deposits, tend to establish new relationships with riskier firms and that these firms increase investment and employment. Bottero et al. (2019) find that in Italy a higher net interbank positions of banks are associated with an increase in total credit and an increase in investment of firms with business relationships to these banks. However, the overall effect of NIRP seems to be not obvious as there is also evidence that firms in existing relationships with German banks, which are more exposed to NIRP, face a contraction in credit supply associated with lower employment (Moser et al. 2021). Finally, a business survey among 500 German firms showed that 32% of those firms confronted with negative deposit interest rates increased their investment (Commerzbank 2019).

Estimates of the ECB suggest a positive but small impact of NIRP on output and inflation. The estimates are based on counterfactual scenarios for interest rates–with and without unconventional policy measures–in small VAR models (Rostagno et al. 2019). They find unconventional measures increased the level of GDP between 2.5% and 3% in the euro area at the end of 2019. The annual inflation rate between 2014 and 2019 was higher by 1/3 to 1/2% points due to unconventional monetary policy (Boucinha and Burlon 2020). One-sixth of this effect can be attributed to NIRP according to the results. Even though the uncertainty surrounding these estimates is high, a moderate positive effect of NIRP on output and inflation seems to be plausible.

Literature on the impact of NIRP on output or inflation expectations is also scarce. Czudaj (2020) uses survey-based expectations data for up to 44 economies to analyse the impact of the adoption of a negative interest rate policy on expectations made by professional forecasters based on a difference-in-differences approach. He finds that the introduction of negative policy rates significantly reduces expectations regarding 3-month money market interest rates and also 10-year government bond yields. He also provides evidence for a significantly positive effect of this unconventional monetary policy tool on GDP growth and inflation expectations.

## NIRP and the general monetary policy enviroment

Negative interest rate policies are not fundamentally different from conventional monetary policy or other unconventional measures, but exhibit specific features. Monetary policy can transmit via various channels to economic activity and inflation, ranging from the impact on credit supply and demand to expectations or exchange rates. Monetary policy instruments address these channels to varying degrees. Compared to other unconventional monetary policy measures, such as forward guidance or quantitative easing, NIRP has a closer link to the bank lending channel via its direct impact on bank profitability and credit supply. In this regard, NIRP is similar to conventional interest rate adjustments when interest rates are in positive territory. However, bank data show that banks are much more reluctant to transmit lower central bank rates to deposits – in particular to charge negative rates on household deposits – than to loan rates. This lowers bank profitability and weighs on credit supply, reducing the effectiveness of NIRP in this regard potentially to the point that a further reduction of the deposit facility rate leads to a contraction of loan supply (reversal interest rate). The strength of this effect depends on different factors, including the role of deposit funding of individual banks or the banking system. NIRP can have larger effects on expectations about the future stance of monetary policy than conventional measures when implemented the first time if market participants did not expect a central bank to use this instrument. However, this feature can be exploited only once and will not take place in subsequent interest rate adjustments. Specific to NIRP is also that negative deposit facility rates can reduce bank profitability, particularly when excess liquidity is high. However, the ECB introduced measures to mitigate this effect and there is so far no conclusive evidence that excess liquidity has become a relevant determinant for bank credit supply. Finally, to the extent that negative interest rates are transmitted to deposit accounts of firms or private household they could have more than proportional effects on risk-taking of firms and private households when they have a high preference to avoid negative interest rates due to psychological effects. However, empirical evidence on such effects is scarce.

Negative interest rates are likely to remain one instrument of the ECB’s monetary policy toolbox, but can be used only to a limited degree to further loosen monetary policy. NIRP is one among several tools that central banks can use to further loosen monetary policy, once the zero lower bound restricts the use of conventional interest rate policies. The experience until now does not point to fundamental differences in terms of its impact as well in terms of its risks compared to other measures, even though some central banks are reluctant to use this instrument. Central banks will prefer to have several unconventional instruments in their toolbox given the high uncertainty with regard to the eventual impact on output as well as inflation of each individual instrument and given that all of these measures can only be implemented up to a certain limit. NIRP faces such limitations as well. The reversal interest rate—the rate at which further declines in interest rates will have a negative impact on lending and thereby counteract the intended effect—cannot be directly observed, but the room for further interest rates cuts of the ECB seems to be small. This is particularly true as the negative effects of NIRP to bank profitability tends to increase the longer NIRP is in place even without further interest rate cuts. Finally, the more negative interest rates will become, the more cash holding could increase, making this instrument less effective, as well.

The effectiveness of monetary policy with respect to output and inflation depends on the economic circumstances and varies over time. The impact of monetary policy on output and prices depends on the specific economic circumstances and varies over the business cycle. There is evidence that monetary policy is particularly effective in crisis times when uncertainty and financial frictions are particularly high (Ciccarelli et al. 2013; Dahlhaus 2017; Jannsen et al. 2019) but less effective in subsequent recovery periods or normal periods (Bech et al. 2014; Jannsen et al. 2019; Burgard et al. 2019). Moreover, there is empirical evidence that expansionary monetary policy has smaller effects in stimulating economic activity than contractionary monetary policy in dampening economic activity (Angrist et al. 2016; Tenreyro and Thwaites 2016), even though the results with regard to the impact on prices are somewhat ambiguous (Deborttoli et al. 2020). Finally, literature reviews suggest that expansionary monetary policy may lose impact the longer it is in place (Borio and Zabai 2016). In addition, the flattening of the Phillips curve has made it more difficult for central banks to influence inflation via aggregate demand. While estimation results on the Phillips curve are very uncertain because it is difficult to disentangle the impact of aggregate demand and other possible factors (e.g., inflation expectations), many studies point to a weakening of the Phillips curve in recent decades (BIS 2017; IMF 2013; Blanchard et al. 2015; Lodge and Mikolajun 2016). Even studies that report a stable relationship find that the relationship is relatively weak (Eser et al. 2020). The weakening of the Phillips curve would be less of a concern for central banks if it was mainly due to better anchored inflation expectations. The better inflation expectations are anchored at central banks inflation targets, the lower the impact on inflation of fluctuations in aggregate demand or other factors. However, when inflation deviates from the inflation target for extended periods a weaker Phillips curve makes it more difficult for central banks to control inflation via influencing aggregate demand. Moreover, a weak or unstable Philips curve makes it more difficult to assess and to forecast the impact of monetary policy measures on inflation, increasing the uncertainty about the appropriate stance of monetary policy.

If the impact of monetary policy on output and inflation depends on the degree of financial frictions, the effects of NIRP have become smaller after their implementation. The empirical evidence suggests that monetary policy is particularly effective in periods of high financial distress and uncertainty but less effective in other periods. One reason could be that in periods of high financial distress and uncertainty access to finance for firms becomes more restrictive and at the same time the need for financing is increasing as these periods are usually associated with weak economic activity and sales. This aspect could be particularly relevant for NIRP, which has a more direct focus on stimulating output and inflation by increasing bank loan supply than other measures, such as asset purchases. Financial distress and uncertainty were high during the global financial crisis and the sovereign debt crises in the euro area – albeit to a varying degree across euro area countries –, but declined to much lower levels afterwards. Survey data of the European Commission suggests that access to finance has not been an important problem for most of the firms in the manufacturing sector since 2014 and by far less of an issue than other problems, such as insufficient demand or availability of skilled staff.^4^ As access to finance was not an important limiting factor to economic activity in recent years, the impact of monetary policy and in particular of NIRP in this period may have been low. Increasing bank loan supply and easing financial conditions via NIRP is not a well-targeted policy if a shortage of loan supply and tight financial conditional are not the underlying reasons for low inflation. In spring 2020, financial conditions considerably tightened due to the COVID-19 pandemic, potentially increasing the impact of monetary accommodation.

From a theoretical perspective, a decline in the natural interest rate makes it more difficult for central banks to stimulate the economy and to control inflation. The natural interest rate is a theoretical concept that describes the equilibrium real interest rate that prevails if GDP is equal to potential output and inflation is equal to the inflation target of the central bank. It has strong implications for monetary policy as it determines the neutral stance when interest rates are equal to the natural rate. The natural rate is closely related to desired savings and desired investment and depends on many factors, such as productivity, demographic change, investment opportunities, or financial integration. The natural rate cannot be observed and therefore has to be estimated. Recent estimates point to a decline of the natural rate in the past decades (Fiedler et al. 2018). This implies that it has become more likely that central banks hit the zero lower bound with their conventional interest rate instruments making a case for unconventional policy measures. Some estimates suggest that the natural rate even has become negative, making an even stronger case for unconventional measures and negative interest rate policies.

Estimates of the natural interest rate are subject to high uncertainty and are not a reliable guidance for monetary policy. While available estimates usually find that the natural interest rate has declined in the past decade, they offer a wider range of point estimates. Many estimates rely on small semi-structural models that use an IS-equation (that links deviations of the interest rate from the natural interest rate to the output gap) and a Phillips curve (that links the output gap to inflation) to identify fluctuations in the natural rate (Holston et al. 2016; Laubach and Williams 2003, 2016). However, these relationships seem to vary over time, which is difficult to empirically account for, increasing estimation uncertainty (Borio 2017). Moreover, the resulting estimates of the natural rate are subject to revisions when additional or revised date become available so that their use in real time, which is key for policy making, is questionable (Clark and Kozicki 2005). The natural rate can also be impacted by other factors, such as the financial cycle (reflected by longer-term fluctuations in credit and housing markets), or the stance of monetary policy itself (Kiley 2015). Estimates that try to account for financial imbalances (Belke and Klose 2020; Juselius et al. 2017) or the monetary policy stance (Taylor and Wieland 2016) sometimes lead to higher estimates of the natural interest rate, even though the results seem to be sensitive to different modelling strategies so that the range of available results is large (Brand and Maelis, 2019; Krustev 2019). Overall, the confidence interval surrounding estimates of the natural rate amounts to several percentage points (Beyer and Wieland 2017; Hamilton et al. 2016; Lubik and Matthes, 2015). While the natural rate may be useful as a theoretical concept that helps to better understand the overall economic environment in which central banks operate, its potential for direct application in monetary policy making is limited.

Monetary policy can have unintended negative side effects that tend to increase the longer expansionary monetary is in place. Monetary policy can have several negative side effects that harm the economy:

- First, expansionary monetary policy leads to increasing risk-taking (Drehmann et al. 2012; Rajan 2005, Maddaloni and Peydro 2011). While increasing risk-taking is one channel through which expansionary monetary policy works to achieve higher economic activity and inflation, excessive risk-taking at the same time can promote financial imbalances and thereby undermine financial stability. Financial risks tend to increase the longer expansionary monetary policy is in place (Kahn 2010; Maddaloni and Peydro 2011). Macroprudential policies may help to contain these risks but are unlikely to avoid all kinds of financial imbalances or financial crises (Adrian 2020).
- Second, expansionary monetary policy can contribute to a misallocation of resources. For example, there is some evidence that lower real interest rates are associated with lower productivity (Cette et al. 2016). One channel is when monetary policy promotes the so-called “zombification”, by enabling not creditworthy firms to receive further funding and stay afloat. In the euro area, the outright monetary transactions (OMT) announcement has led to an increase of below average credit servicing ability (Acharya et al. 2019). The phenomenon has been observed also in Japan (Caballero et al. 2008; Hoshi and Kashyap 2004). Even though the link is theoretically plausible and there is some evidence that lower interest rates are associated with a higher degree of zombification (Banerjee and Hofmann 2018), it is difficult to empirically identify a direct link between monetary policy and zombification.
- Third, the longer a very expansionary monetary policy is in place, the more difficult it will become for central banks to tighten monetary policy at the same pace as it was usually observed before the global financial crisis because the impact and therefore the risks for turmoil on financial markets and sovereign debt markets are increasing, the longer low interest rates are in place. As a consequence, central banks may lose credibility with respect to their determination to fight inflation once it rises and threatens to violate the goal of maintaining price stability because it becomes more likely that they will tolerate higher inflation to avoid financial market turmoil. Monetary tightening is particularly tricky when government finances are built on artificially low long-term interest rates, like currently in a number of euro area countries, may put the independence of central banks at risk (Fiedler et al. 2020). Relatedly, expansionary monetary policy also decreases the incentives for structural reforms of governments.

All of these side effects of monetary policy are difficult to quantify– even ex-post, and it is of course also difficult to assess the specific side effects of NIRP. To the extent that NIRP has more than proportional effects on risk-taking, the side effects from higher risk-taking should be larger. Overall, the general stance of monetary policy seems to be more important for side effects than specific policy measures. However, incentives for central banks to take these not directly observable side effects into account when conducting monetary policy are relatively low given that their policy is evaluated mainly against their exactly defined inflation target.

Questions regarding the effectiveness of monetary policy, potential side effects of low for long policies and the monetary policy strategy in general are more relevant for the ECB than the question whether to use NIRP as one of many instruments at the current juncture. Many central banks have missed their inflation target for extended periods of time after the global financial crisis and for longer periods than they were used to before. Empirical evidence suggests that one reason behind could be lower effectiveness of monetary policy in stimulating output and inflation. As a consequence, central banks loosened their policies to unprecedented levels and maintained an expansionary stance of their policy for a very long period of time.

## Conclusions

This paper has argued that NIRP had some positive impact on loan growth and investment in the euro area, but that the room to further loosen monetary policy via NIRP may be small. While negative economic side effects of expansionary monetary policy are difficult to identify and to quantify, it seems natural to assume that the risks for such side effects increase the more expansionary monetary policy is and the longer such policy is in place. The strong increase in inflation has increased the pressure on the ECB to change the path of monetary policy and abandoning the negative interest policy. Against this backdrop, it seems more important for the ECB (and other central banks) to discuss whether its monetary strategy has to be adjusted to these new circumstances than to discuss whether specific measures, such as NIRP, are appropriate to conduct monetary policy within this strategy.

## Footnotes

1 Repullo (2020) proposes an alternative model in which the bank’s equity capital is not exogenous. The future value of the bank’s capital is driven by two components: profits from lending, equal to the spread between the loan rate and the policy rate multiplied by the total amount of loans, and profits from deposit taking, equal to the spread between the policy rate and the deposit rate multiplied by the total amount of deposits.

2 Urbschat (2018) documents that deposit-reliant banks in Germany have lower net interest margins when policy rates become negative.

3 Shadow rates as for example adopted by Wu and Xia (2016) are also considered for assessing the impact of unconventional monetary policy on the term structure on interest rates and macroeconomic outcomes. However, their fluctuations are not associated with changes in the deposit rate. Overall, a shortcoming of shadow rates is that they are generated regressors which are quite sensitive with regard to underlying modelling and estimation choices (Krippner 2019). This makes the identification of effects on output and inflation very demanding. Krippner (2019) shows that, depending on the modelling choice, effects on inflation and unemployment can vary up to 2.21 and 1.80 basis points.

4 Survey data on limits to production in the service industry are only available since 2003. On average, in the service industry more firms report about financial conditions to limit their production. However, the share of firms in the service industry reporting from such problems in 2014 (15,6%) was only slightly higher than in the period from 2013 to 2017 (14%) and declined until 2020.

## References

[CR1] Acharya VV, Eisert T, Eufinger C, Hirsch C (2019) Whatever it takes: The real effects of unconventional monetary policy. Rev Financ Stud 32(9):3366–3411

[CR2] Adrian T (2020) Low for long’ and risk-taking. Monetary and Capital Markets Department (Series) No 20/15. https://www.imf.org/en/Publications/Departmental-Papers-Policy-Papers/Issues/2020/11/23/Low-for-Long-and-Risk-Taking-49733

[CR3] Adolfsen JF, Spange M (2020) Modest pass-through of monetary policy to retail rates but no reversal. Danmarks Nationalbank Working Papers No 154. https://www.econstor.eu/bitstream/10419/227872/1/1694096718.pdf

[CR4] Angrist JD, Jordá Ó, Kuersteiner GM (2016) Semiparametric estimates of monetary policy effects: string theory revisited. J Bus Econ Stat 36(3):371–387

[CR5] Altavilla C, Boucinha M, Motto R (2015) Asset purchase programmes and financial markets: evidence from the euro area. ECB Working Paper No. 1864. https://www.ecb.europa.eu/pub/pdf/scpwps/ecbwp1864.en.pdf

[CR6] Altavilla C, Boucinha M, Peydró J-L (2018) Monetary policy and bank profitability in a low interest rate environment. Econ Policy 33, pp 531–586. See also ECB Working Paper Series, No 2105. https://www.ecb.europa.eu/pub/pdf/scpwps/ecb.wp2105.en.pdf?6b8a3f70b10e04798981cbe109df411e

[CR7] Altavilla C, Burlon L, Giannetti M, Holton S (2019) Is there a zero lower bound? The effects of negative policy rates on banks and firms. ECB Working Paper Series, No 2289. https://www.ecb.europa.eu/pub/pdf/scpwps/ecb.wp2289~1a3c04db25.en.pdf

[CR8] Ampudia M, Van den Heuvel S (2018) Monetary policy and bank equity values in a time of low interest rates”. ECB Working Paper No 2199. See also ECB Research Bulletin No 60. https://www.ecb.europa.eu/pub/pdf/scpwps/ecb.wp2199.en.pdf?c5c651eb06e94cc4d080700b6ff3a11f

[CR9] Amzallag A, Calza A, Georgarakos D, Sousa J (2019) Monetary policy transmission to mortgages in a negative interest rate environment. ECB Working Paper No. 2243. https://www.econstor.eu/bitstream/10419/208277/1/1066618348.pdf

[CR10] Arce Ó, García-Posada M, Mayordomo S(2019) Adapting lending policies against a background of negative interest rates. Econ Bull, Banco de España, issue 1, pages 1–9. https://ideas.repec.org/a/bde/journl/y2019i3daan5.html

[CR11] Arce O, García-Posada M, Mayordomo S, Ongena S (2018) Adapting lending policies when negative interest rates hit banks’ profits. Working Papers, No. 1832, Banco de España. https://www.bde.es/f/webbde/SES/Secciones/Publicaciones/PublicacionesSeriadas/DocumentosTrabajo/18/Files/dt1832e.pdf

[CR12] Bank for International Settlements (BIS) (2017) Monetary policy: Inching towards normalisation. 87th Annual Report, 2016/17, Chap. 4. Available at: https://www.bis.org/publ/arpdf/ar2017e4.htm

[CR13] Banerjee RN, Hofmann B (2018) The rise of zombie firms: causes and consequences. BIS Quarterly Review September: 67–78. Available at: https://www.bis.org/publ/qtrpdf/r_qt1809g.pdf

[CR14] Basten C, Mariathasan M (2018) How banks respond to negative interest rates: Evidence from the Swiss exemption threshold. CESifo Working Paper Series 6901. https://papers.ssrn.com/sol3/papers.cfm?abstract_id=3164780

[CR15] Beauregard R, Spiegel MM (2020) Commercial banks under persistent negative rates. FRBSF Economic Letter 2020-29. https://www.frbsf.org/economic-research/publications/economic-letter/2020/september/commercial-banks-under-persistent-negative-rates/

[CR16] Bech M, Gambacorta L, Kharroubi E (2014) Monetary policy in a downturn: are financial crises special? Int Financ 17(1):99–119

[CR17] Beck R, Duca IA, Stracca L (2019) Medium term treatment and side effects from quantitative easing: International evidence. ECB Working Paper No. 2229. https://www.ecb.europa.eu/pub/pdf/scpwps/ecb.wp2229~00d920df20.en.pdf

[CR18] Belke A, Klose J (2020) Equilibrium real interest rates and the financial cycle: empirical evidence for euro area member countries. Econ Model 84:357–366

[CR19] Beyer R, Wieland V (2017) Instability, imprecision and inconsistent use of equilibrium real interest rate estimates. J Int Money Financ 94:1–14

[CR20] Bittner C, Bonfim D, Heider F, Saidi F, Schepens G, Soares C (2020) Why so negative? The effect of monetary policy on bank credit supply across the euro area. unpublished working paper

[CR21] Blanchard O, Cerutti E, Summers L (2015) Inflation and activity – two explorations and their monetary policy implications. NBER Working Papers 21726. Cambridge, MA. https://www.nber.org/papers/w21726

[CR22] Borio C, Zhu H (2008) Capital regulation, risk-taking and monetary policy: a missing link in the transmission mechanism?. BIS Working Papers No 268. https://www.bis.org/publ/work268.pdf

[CR23] Borio C, Zabai A(2016) Unconventional monetary policies: a re-appraisal. BIS Working Papers 570. Available at: http://www.bis.org/publ/work570.pdf

[CR24] Borio C, Gambacorta L (2017) Monetary policy and bank lending in a low interest rate environment: Diminishing effectiveness? Journal of Macroeconomics 54(PB):217–231

[CR25] Bottero M, Minoiu MC, Peydró JL, Polo A, Presbitero MAF, Sette E (2019) Negative monetary policy rates and portfolio rebalancing: Evidence from credit register data. IMF Working Paper No. 19 /44. https://www.imf.org/en/Publications/WP/Issues/2019/02/28/Negative-Monetary-Policy-Rates-and-Portfolio-Rebalancing-Evidence-from-Credit-Register-Data-46638

[CR26] Boucinha M, Burlon L (2020) Negative rates and the transmission of monetary policy. ECB Economic Bulletin, Issue 3/2020. https://www.ecb.europa.eu/pub/pdf/ecbu/eb202003.en.pdf

[CR27] Brand C, Mazelis F (2019) Taylor-rule consistent estimates of the natural rate of interest. ECB Working Paper Series 2257. Available at: https://www.ecb.europa.eu//pub/pdf/scpwps/ecb.wp2257~b842f47cf9.en.pdf

[CR28] Brunnermeier M, Koby Y (2018) The Reversal Interest Rate. NBER Working Paper No 25406. https://www.nber.org/system/files/working_papers/w25406/w25406.pdf

[CR29] Bubeck J, Maddaloni A, Peydró J-L (2020) Do banks invest in riskier securities in response to negative central bank interest rates?. ECB Research Bulletin No 70. https://www.ecb.europa.eu/pub/economic-research/resbull/2020/html/ecb.rb200421~c06c3ed3c0.en.html

[CR30] Burgard J-P, Neuenkirch M, Nöckel M (2019) State-dependent transmission of monetary policy in the euro area. J Money Credit Bank 51(7):1733–2072

[CR31] Caballero RJ, Hoshi T, Kashyap A (2008) Zombie lending and depressed restructuring in Japan. Am Econ Rev 98(5):1943–1977

[CR32] Carbó-Valverde S, Cuadros-Solas P, Rodriguez-Fernández F (2021) The effects of negative interest rates: a literature review and additional evidence on the performance of the European banking sector. Eur J Financ

[CR33] Cette G, Fernald J, Mojon B (2016) The pre-great recession slowdown in productivity. Eur Econ Rev 88:3–20

[CR34] Ciccarelli M, Maddaloni A, Peydro J-L (2013) Heterogeneous transmission mechanism: monetary policy and financial fragility in the eurozone. Econ Policy 28(75):459–512

[CR35] Clark T, Kozicki S (2005) Estimating equilibrium real interest rates in real time. North Am J Econ Financ 16(3):395–413

[CR36] Commerzbank (2019) Zwischen Sicherheit und Chance: Wie der Mittelstand anlegt. Marktstudie der Commerzbank in Zusammenarbeit mit forsa. https://www.firmenkunden.commerzbank.de/portal/media/corporatebanking/neu-hauptportal-rebrush/insights/anlagestudie/2019_10_Anlagestudie2019_Commerzbank_Forsa.pdf

[CR37] Czudaj RL (2020) Is the negative interest rate policy effective? J Econ Behav Organ 174(C):75–86

[CR38] Dahlhaus T (2017) Conventional monetary policy transmission during financial crises: an empirical analysis. J Appl Econ 32:401–421

[CR39] Darracq Pariès M, Kok C, Rottner M (2020) Reversal interest rate and macroprudential policy. ECB Working Paper, No. 2487. https://www.ecb.europa.eu/pub/pdf/scpwps/ecb.wp2487~77052f3728.en.pdf

[CR40] De Groot O, Haas A(2019) The signalling channel of negative interest rates. MPRA Paper No.95479. https://mpra.ub.uni-muenchen.de/95479/1/MPRA_paper_95479.pdf

[CR41] Debortoli D, Forni M, Gambetti L, Sala L (2020) asymmetric effects of monetary policy easing and tightening. CEPR Discussion Papers 15005

[CR42] Demiralp S, Eisenschmidt J, Vlassopoulos T (2019) Negative interest rates, excess liquidity and bank business models: Banks’ reaction to unconventional monetary policy in the euro area”. ECB Working Paper no. 2283. https://www.ecb.europa.eu/pub/pdf/scpwps/ecb.wp2283~2ccc074964.en.pdf?fbb6d4de645fdd3ea2f6b24834bfd82c

[CR43] Drehmann M, Borio C, Tsatsaronis K (2012) Characterising the financial cycle: don’t lose sight of the medium term! BIS Working Paper 380. https://www.bis.org/publ/work380.pdf

[CR44] Eggertsson G, Juelsrud R, Summers L, Getz Wold E (2019) Negative nominal interest rates and the bank lending channel. NBER Working Paper Series, No 25416. https://www.nber.org/system/files/working_papers/w25416/w25416.pdf

[CR45] Eser F, Karadi P, Lane PR, Moretti L, Osbat C (2020) The Phillips Curve at the ECB. ECB Working Paper Series 2400. https://www.econstor.eu/bitstream/10419/229014/1/ecb-wp2400.pdf

[CR46] Fiedler S, Gern K-J, Jannsen N, Wolters M (2018) Growth prospects, the natural interest rate, and monetary policy. In-Depth Analysis for the European Parliament, Policy Department A: Economic and Scientific Policy, Monetary Dialogue Papers, November 2018. https://www.europarl.europa.eu/cmsdata/157015/KIEL%20final%20publication.pdf

[CR47] Fiedler S, Gern K-L, Stolzenburg U (2020) Blurred boundaries between monetary and fiscal policy. Briefing paper requested by the European Parliament’s Committee on Economic and Monetary Affairs, Monetary Dialogue Papers, November 2020. https://www.europarl.europa.eu/RegData/etudes/IDAN/2020/658194/IPOL_IDAN(2020)658194_EN.pdf

[CR48] Gagnon J, Raskin M, Remache J, Sack B (2010) Large-scale asset purchases by the federal reserve: Did they work?. FRB of New York Staff Report 441. https://www.newyorkfed.org/medialibrary/media/research/staff_reports/sr441.pdf

[CR49] Grandi P, Guille M (2021) The upside down: banks, deposits and negative rates. Available at SSRN: https://ssrn.com/abstract=3363743 or 10.2139/ssrn.3363743

[CR50] Hamilton JD, Harris E, Hatzius J, West K (2016) The equilibrium real funds rate: past, present, and future. IMF Econ Rev 64(4):660–707

[CR51] Heider F, Saidi F, Schepens G (2019) Life below zero: Bank lending under negative policy rates. Rev Financ Stud 32:3728–3761. 10.1093/rfs/hhz016

[CR52] Holston K, Laubach T, Williams JC (2016) Measuring the natural rate of interest: international trends and determinants. J Int Econ 108:S59–S75. https://www.sciencedirect.com/science/article/pii/S0022199617300065?via%3Dihub

[CR53] Hong GH, Kandrac J (2018) Pushed past the limit? How Japanese banks reacted to negative interest rates. IMF Working Paper No. 18/131. https://www.imf.org/en/Publications/WP/Issues/2018/06/13/Pushed-Past-the-Limit-How-Japanese-Banks-Reacted-to-Negative-Interest-Rates-45927

[CR54] Hoshi T, Kashyap A (2004) Japan‘s financial crisis and economic stagnation. J Econ Perspect 18(1):3–26. 10.1257/089533004773563412

[CR55] International Monetary Fund (IMF) (2013) The dog that didn’t bark: Has inflation been muzzled or was it just sleeping? World Economic Outlook, April, Chap. 3. https://www.imf.org/external/pubs/ft/weo/2013/01/

[CR56] International Monetary Fund (IMF) (2017) Negative interest rate policies—Initial experiences and assessments. IMF Policy Paper. https://www.imf.org/en/Publications/Policy-Papers/Issues/2017/08/03/pp080317-negative-interest-rate-policies-initial-experiences-and-assessments

[CR57] Jannsen N, Potjagailo G, Wolters M (2019) Monetary policy during financial crises: is the transmission mechanism impaired? Int J Cent Bank 15(4):81–126

[CR58] Juselius M, Borio C, Disyatat P, Drehmann M (2017) Monetary policy, the financial cycle, and ultra-low interest rates. Int J Cent Bank 13(3):55–89

[CR59] Kahn GA (2010) Taylor rule deviations and financial imbalances. Econ Rev (2):63–99

[CR60] Kiley MT (2015) What can the data tell us about the equilibrium real interest rate?. Finance and Economics Discussion Series 2015-077. Board of Governors of the Federal Reserve System. https://www.federalreserve.gov/econresdata/feds/2015/files/2015077pap.pdf

[CR61] Klein M (2020) Implications of negative interest rates for the net interest margin and lending of euro area banks. Deutsche Bundesbank Discussion Paper, No. 10/2020. https://www.bundesbank.de/en/publications/research/discussion-papers/implications-of-negative-interest-rates-for-the-net-interest-margin-and-lending-of-euro-area-banks-828040

[CR62] Krippner L (2019) A note of caution on shadow rate estimates. J Money Credit Bank 52(4):951–962

[CR63] Krogstrup S, Kuchler A, Spange M (2020) Negative interest rates: The Danish experience. VOXEU blog post, 2. October 2020. https://voxeu.org/article/negative-interest-rates-danish-experience

[CR64] Krustev G (2019) The natural rate of interest and the financial cycle. J Econ Behav Control 162:193–210

[CR65] Laubach T, Williams JC (2003) Measuring the natural rate of interest. Rev Econ Stat 85:1063–1070

[CR66] Laubach T, Williams C (2016) Measuring the natural rate of interest redux. Business Economics 51:57–67. 10.1057/be.2016.23

[CR67] Lopez JA, Rose AK, Spiegel MM (2020) Why have negative nominal interest rates had such a small effect on bank performance? Cross country evidence. Eur Econ Rev 124:103402

[CR68] Lodge D, Mikolajun I (2016) Advanced economy inflation: The role of global factors. ECB Working Paper no 1948. 10.2139/ssrn.2831946

[CR69] Lubik T, Matthes C (2015) Calculating the natural rate of interest: A comparison of two alternative approaches. Richmond Fed Economic Brief EB15-10. https://www.richmondfed.org/publications/research/economic_brief/2015/eb_15-10

[CR70] Madaschi C, Nuevo IP (2017) The profitability of banks in a context of negative monetary policy rates: the cases of Sweden and Denmark. ECB Occasional Paper Series no.195. https://www.ecb.europa.eu/pub/pdf/scpops/ecb.op195.en.pdf

[CR71] Maddaloni A, Peydró J-L (2011) Bank risk-taking, securitization, supervision, and low interest rates: Evidence from the Euro-area and the U.S. lending standards. Rev Financ Stud 24(6):2121–2165. 10.1093/rfs/hhr015

[CR72] Michail NA (2019) What if they had not gone negative? A counterfactual assessment of the impact from negative interest rates. Oxf Bull Econ Stat 81:1–19

[CR73] Molyneux P, Reghezza A, Xie R (2019) Bank margins and profits in a world of negative rates. J Bank Financ 107(C):1–1

[CR74] Moser C, Saidi F, Wirth B, Wolter S (2021) Credit supply, firms, and earnings inequality. Econtribute Discussion Paper 086. https://www.econtribute.de/RePEc/ajk/ajkdps/ECONtribute_086_2021.pdf

[CR75] Paligorova T, Sierra Jimenez JA (2012) Monetary policy and the risk-taking channel: insights from the lending behaviour of banks. Bank of Canada Review, vol 2012(Autumn): 23–30. https://www.bankofcanada.ca/wp-content/uploads/2012/11/boc-review-autumn12-paligorova.pdf

[CR76] Repullo R (2020) The reversal interest rate. A critical review. CEMFI Working Papers no. 2021. https://www.cemfi.es/ftp/wp/2021.pdf

[CR77] Rajan R (2005) Has financial development made the world riskier? NBER Working Paper No. 11728. 10.3386/w11728

[CR78] Rostagno M, Altavilla C, Carboni G, Lemke W, Motto R, Saint Guilhem A, Yiangou J (2019) A tale of two decades: the ECB’s monetary policy at 20. ECB Working Paper Series 2346. https://www.ecb.europa.eu/pub/pdf/scpwps/ecb.wp2346~dd78042370.en.pdf

[CR79] Ryan E, Whelan K (2021) Quantitative easing and the hot potato effect: Evidence from Euro Area Banks. J Int Money Financ 115

[CR80] Schelling T, Towbin P (2018) Negative interest rates, deposit funding and bank lending. SNB Working Paper No. 5/2020. https://www.snb.ch/n/mmr/reference/working_paper_2020_05/source/working_paper_2020_05.n.pdf

[CR81] Schnabel I (2020) Going negative: the ECB’s experience. Speech at the Roundtable on Monetary Policy, Low Interest Rates and Risk Taking at the 35th Congress of the European Economic Association. https://www.ecb.europa.eu/press/key/date/2020/html/ecb.sp200826~77ce66626c.en.html

[CR82] Stráský J, Hwang H (2019) Negative interest rates in the euro area: does it hurt banks?. OECD Economics Department Working Papers 1574, OECD Publishing. https://www.oecd-ilibrary.org/docserver/d3227540-en.pdf?expires=1622664472&id=id&accname=guest&checksum=480D2D3AC5259825D739CBC1402CC7FF

[CR83] Tagesschau (2020) Strafzinsen treffen viele Kunden. 3.4.2021. https://www.tagesschau.de/wirtschaft/finanzen/banken-kontofuehrungsgebuehren-strafzinsen-101.html

[CR84] Tan G (2019) Beyond the zero lower bound: negative policy rates and bank lending. DNB Working Paper No. 649. https://www.dnb.nl/media/jdahu2g3/working-paper-no-649_tcm47-385679.pdf

[CR85] Taylor J, Wieland V (2016) Finding the equilibrium real interest rate in a fog of policy deviations. Business Economics 51:147–154. 10.1057/s11369-016-0001-5

[CR86] Tenreyro S (2021) Let’s talk about negative rates. Speech given at UWE Bristol Webinar on 11 January 2021. https://www.bankofengland.co.uk/speech/2021/january/silvana-tenreyro-lets-talk-about-negative-interest-rates

[CR87] Tenreyro S, Thwaites G (2016) Pushing on a string: US monetary policy is less powerful in recessions. Am Econ J Macroecon 8(4):43–74. 10.1257/mac.20150016

[CR88] Urbschat F (2018) The good, the bad, and the ugly: Impact of negative interest rates and QE on the profitability and risk-taking of 1600 German Banks". CESifo Working Paper No. 7358. 10.2139/ssrn.3338686

[CR89] Wu JC, Xia FD (2016) Measuring the macroeconomic impact of monetary policy at the zero lower bound. J Money Credit Bank 48:253–291. 10.1111/jmcb.12300

[CR90] Ziegler-Hasiba E, Turnes E (2018) Negative interest rate policy in Switzerland. Entrep Bus Econ Rev 6(3):103–128

